# Quality of Life, Caregiver Burden, and Symptoms of Depression and Anxiety in Parents of Children with Spinal Muscular Atrophy: A Comparison with Healthy Controls

**DOI:** 10.3390/medicina61050930

**Published:** 2025-05-21

**Authors:** Zehra Koyuncu, Seda Sönmez Kurukaya, Fitnat Uluğ, Tuğçe Damla Dilek, Yılmaz Zindar, Büşra Arslan, Berkay Tayşi, Elif Anaç, Mustafa Balkanas, Sena Kesik, Kevser Sak, Ömer Faruk Demirel, Burak Doğangün, Sema Saltık

**Affiliations:** 1 Department of Child and Adolescent Psychiatry, Cerrahpasa Faculty of Medicine, Istanbul University-Cerrahpasa, 3409 Istanbul, Turkey; drberkaytaysi@gmail.com (B.T.); dr.elif.anac.1994@gmail.com (E.A.); balkanas.96@gmail.com (M.B.); kesik.sena@gmail.com (S.K.); burakdogangun@hotmail.com (B.D.); 2 Department of Pediatric Neurology, Cerrahpasa Faculty of Medicine, Istanbul University-Cerrahpasa, 3409 Istanbul, Turkey; sedasnmz@gmail.com (S.S.K.); yilmazzindar@gmail.com (Y.Z.); saltiksema@gmail.com (S.S.); 3 Department of Pediatric Neurology, Istanbul Prof. Dr. Cemil Taşcioğlu City Hospital, 34020 Istanbul, Turkey; fitnatulug76@gmail.com; 4 Departmant of Pediatrics, Södra Alvsborgs Hospital, 50182 Borås, Sweden; drtugcedamla@gmail.com; 5 Child and Adolescent Psychiatry Clinic, Amasya Sabuncuoglu Serefeddin Research and Trainee Hospital, 05000 Amasya, Turkey; md.busraarslan@gmail.com; 6 Dulkadiroğlu District Health Directorate, 46050 Kahramanmaras, Turkey; kevser.sak@saglik.gov.tr; 7 Department of Psychiatry, Cerrahpasa Faculty of Medicine, Istanbul University-Cerrahpasa, 3409 Istanbul, Turkey; ofdmed@yahoo.com

**Keywords:** SMA, parent, quality of life, caregiver burden, depression, anxiety

## Abstract

*Background*: Spinal muscular atrophy (SMA) is a disease that leads to muscle weakness and significantly affects the lives of both patients and caregivers. This study aims to compare quality of life, caregiver burden, symptoms of depression and anxiety, life satisfaction, and mental well-being between parents of children with SMA and those of healthy children. *Methods*: This cross-sectional study included parents of children under 18 years old, both healthy and diagnosed with SMA. The participants completed the WHOQOL-BREF, Zarit Caregiver Burden Scale (ZCBS), Hospital Anxiety and Depression Scale (HADS), Satisfaction with Life Scale (SWLS), and the Flourishing Scale (FS). In addition, the relationships among these measures were analyzed. *Results*: Our study showed that the parents of children with SMA had significantly higher scores across all subscales of the WHOQOL-BREF (*p* = 0.004, *p* = 0.009, *p* = 0.007, *p* < 0.001) and the HADS depression subscale (*p* = 0.005). However, no significant differences were found between the groups in terms of the ZCBS, the HADS anxiety subscale (*p* = 0.802), SWLS (*p* = 0.251), or FS (*p* = 0.929) scores. Additionally, the ZCBS and HADS anxiety scores were significantly higher among parents of children with SMA type 1 compared to those with type 3 (*p* = 0.010 and *p* = 0.037, respectively). Lastly, a moderate positive correlation was found between the ZCBS and the HADS anxiety subscales (r = 0.632, *p* < 0.001). *Conclusions*: This study highlights the decline in quality of life and increased depression symptoms among parents of children with SMA, suggesting the need for psychiatric evaluation and additional support for those caregivers.

## 1. Introduction

Health-related quality of life (HRQOL) is a multidimensional construct defined by the World Health Organization as encompassing physical health, psychological well-being, level of independence, social relationships, personal beliefs, and interactions with salient features of the environment [1]. In the context of chronic medical or neurological conditions, the provision of care is frequently undertaken by informal caregivers, typically family members or close acquaintances, to address the ongoing medical and daily living needs of affected individuals. The assumption of such caregiving roles, which are often prolonged and resource-intensive, has been associated with heightened levels of psychological distress and caregiver burden [2,3]. This burden, in turn, may contribute to a deterioration in caregivers’ own quality of life and psychological health and has been linked to an increased risk of developing psychiatric morbidities [3].

Spinal muscular atrophy (SMA) is an autosomal recessive neurodegenerative disorder that arises due to mutations in both alleles of the survival motor neuron (SMN) gene located on chromosome 5q13.2. Due to its progressive nature, SMA often leads to muscle weakness, paralysis, and death if left untreated. Clinically, SMA is classified into types 0 through 4, based on the onset of symptoms and acquired motor skills. In the most common form, SMA type 1 symptoms typically begin before 6 months of age, and the majority of untreated patients do not survive beyond 2 years [4].

In recent years, the U.S. Food and Drug Administration (FDA) has approved three therapeutic agents—nusinersen, onasemnogene abeparvovec, and risdiplam—for the treatment of SMA. Each targets an increase in SMN protein expression, though via distinct mechanisms of action [5]. Among patients with SMA, the administration of these therapies, particularly when initiated in the early stages of the disease, has been associated with significant improvements in both survival rates and functional outcomes [6]. Additionally, supportive care is frequently necessary due to secondary complications resulting from muscle weakness, including respiratory and nutritional difficulties, as well as orthopedic problems [5]. In many countries, newborn screening programs for SMA are conducted to initiate treatment during the presymptomatic period [7]. Despite the availability of effective treatment options, a significant number of patients face difficulties accessing medications and support systems due to organizational and financial reasons.

Previous research has indicated that caregivers of children with SMA, who require continuous care from birth, report extensive time dedicated to caregiving, disruptions in personal and professional life, decreased quality of life, and increased economic and social burdens [8]. Caregiver burden and quality of life may vary depending on factors such as the SMA subtype [8], access to treatment [9], and the extent of parental engagement in social and leisure activities [10]. Moreover, between 64% and 85% of caregivers and parents of children with SMA report experiencing symptoms of anxiety and depression [10,11].

To the best of our knowledge, previous descriptive studies in this field have not included healthy control groups. Furthermore, no study conducted in Turkey has examined the parents of individuals with SMA in relation to quality of life, caregiver burden, or symptoms of anxiety and depression. Therefore, the present study aimed to (1) compare quality of life, caregiver burden, symptoms of depression and anxiety, life satisfaction, and mental well-being between parents of children with spinal muscular atrophy and parents of healthy children; (2) compare these parameters among parents of children with SMA types 1, 2, and 3; and (3) examine the associations among these variables within the group of parents of children with SMA. The hypotheses of this study were:

1. Caregiver burden and symptoms of depression and anxiety would be higher, while quality of life, life satisfaction, and mental well-being would be lower, in parents of children with SMA compared to parents of healthy children.
2. Caregiver burden and symptoms of depression and anxiety would be higher, while quality of life, life satisfaction, and mental well-being would be lower, in parents of children with SMA type 1 compared to parents of children with SMA types 2 and 3.
3. There would be negative associations between quality of life and symptoms of depression and anxiety in parents of children with SMA. In addition, there would be negative associations between quality of life and caregiver burden in parents of children with SMA.

## 2. Materials and Methods

This cross-sectional, survey-based study compared parents of children diagnosed with SMA to parents of healthy children.

### 2.1. Participants

The study population consisted of parents of children aged 0 to 18 years diagnosed with SMA who were receiving follow-up and treatment at the Pediatric Neurology Clinic of Istanbul University-Cerrahpasa (IU-C), Cerrahpasa Faculty of Medicine. The control group included volunteer parents of children without any diagnosis of chronic illness.

Inclusion criteria for the case group were:

- Having a child aged between 0 and 18 and with the diagnosis of SMA;
- Being literate.

Inclusion criteria for the control group were:

- Having a child aged between 0 and 18 and without any chronic disease;
- Being literate.

Exclusion criteria for the study for both the case and control groups were:

- Having a chronic disease diagnosis in the parent that may cause physical limitations, significant pain, or substantial impacts on daily life (such as rheumatological, orthopedic diseases or cardiac failure);
- Having another child or adult with a chronic illness under the parent’s care.

A total of 65 parents of children with SMA were initially approached for participation in the case group, and 43 consented to take part in the study. None of the participating parents had previously received any form of psychosocial intervention. One parent was excluded due to the death of their child during the study period. Thus, the final case group consisted of 42 parents. All the children in this group were receiving nusinersen treatment. Based on age of onset, symptom severity, and achieved motor milestones, the children were classified into three SMA types [12]. According to this classification, 16 children were diagnosed with SMA type 1, 13 with type 2, and 13 with type 3.

For the control group, 115 parents of children aged 0 to 18 years without chronic illnesses volunteered to participate. From this pool, 42 parents were selected and matched to the case group based on their children’s age and sex.

In summary, the final sample included 42 parents of children with SMA in the case group and 42 age- and sex-matched parents of healthy children in the control group.

### 2.2. Procedure

This study was conducted in the Pediatric Neurology Clinic of Istanbul University-Cerrahpasa (IU-C), Cerrahpasa Faculty of Medicine. Data collection took place between June 2024 and September 2024. Parents were recruited during follow-up visits conducted approximately three months after the administration of nusinersen, with recruitment facilitated by the primary clinicians overseeing the children’s care. The aims and the methods of the study were explained to the parents by the researchers from psychiatry and child and adolescent psychiatry departments. The parents who agreed to participate in the study were asked to complete self-reported scales. A private room was made available to ensure confidentiality and comfort during the completion of the scales. Additionally, they were provided with information on how to contact the researcher if they had any questions. The type of the disease was determined by clinicians’ examination.

The study received ethical approval from the Clinical Research Ethics Committee of IU-C, Cerrahpasa Faculty of Medicine (Approval No: 1017033, dated 14 June 2024). Written informed consent was obtained from all participants prior to their inclusion in the study.

### 2.3. Assessment of Parents

#### 2.3.1. Sociodemographic Characteristics

Sociodemographic characteristics were collected using a researcher-developed form that included questions regarding the child’s and parent’s age, educational level, income status, occupation, medical diagnosis, and marital status.

#### 2.3.2. Health-Related Quality of Life (HRQOL)

Quality of life is defined as an individual’s perception of their position in life in the context of the culture and value systems in which they live and in relation to their goals, expectations, standards, and concerns [13]. The abbreviated version of the World Health Organization Quality of Life Scale (WHOQOL-BREF) which is used in the adult population for a general measurement of quality of life, consists of 26 items. The scale evaluates physical, psychological, social, and environmental domains, with higher scores indicating an improvement in quality of life [14]. It has been widely used to evaluate quality of life in both patients and caregivers affected by neurological disorders, including SMA [15,16].

#### 2.3.3. Caregiver Burden

In our study, the Zarit Caregiver Burden Scale (ZCBS) is used to assess caregiver burden. This 22-item Likert-type scale evaluates various dimensions of caregiving, including health, financial strain, social life, and interpersonal relationships. Higher scores indicate a greater level of distress experienced by the caregiver [17]. The Turkish validation study was conducted by Özlü et al., reporting a Cronbach’s alpha of 0.83, indicating high internal consistency [18].

#### 2.3.4. Symptoms of Depression and Anxiety

In the present study, the validated Turkish version of the Hospital Anxiety and Depression Scale (HADS) was used to assess anxiety and depressive symptoms through self-report. The HADS is one of the most widely used instruments for evaluating psychological distress in hospital settings. The Turkish validation study established cutoff scores of 10 for the anxiety subscale and 7 for the depression subscale [19]. The scale consists of 14 Likert-type items, divided equally between the anxiety and depression subscales [20]. The HADS has been used in previous studies to assess the symptoms among relatives and caregivers of individuals with neurodegenerative diseases [21,22].

#### 2.3.5. Life Satisfaction

Life satisfaction refers to an individual’s cognitive assessment of their quality of life based on their own established standards [23]. The Satisfaction with Life Scale (SWLS), has been used in the literature for caregivers of patients with neurological conditions such as multiple sclerosis and Parkinson’s disease [24,25]. In its Turkish adaptation, the SWLS has been demonstrated to be valid and reliable for use with adults, with a Cronbach’s alpha internal consistency coefficient of 0.88 and a test–retest reliability coefficient of 0.97 [26]. On a scale ranging from 5 to 35, the average individual (50th percentile) scores in the ‘Slightly Satisfied’ (21–25) range, whereas most individuals in mental health settings score in the ‘Slightly Dissatisfied’ (15–19) range [27,28].

#### 2.3.6. Well-Being

The Psychological Well-Being (Flourishing) Scale is an 8-item instrument that assesses psychological well-being across six dimensions: autonomy, environmental mastery, personal growth, positive relationships, purpose in life, and self-acceptance [29]. Each item on the scale is rated between 1 and 7 points (1 = I strongly disagree; 7 = I strongly agree), with higher scores indicating that the individual possesses a greater number of psychological resources and strengths. The factor loadings of the scale items range between 0.61 and 0.77. The Cronbach’s alpha internal consistency coefficient of the scale was found to be 0.87 [30].

### 2.4. Statistical Analysis

Statistical analysis was performed via IBM SPSS Statistics ver. 29 (IBM Corp., Armonk, NY, USA). The Kolmogorov‒Smirnov test was used to evaluate normality. Continuous variables are expressed as medians and interquartile ranges (Q1–Q3), and categorical variables are expressed as frequencies (*n*) and percentages (%). For continuous data, two-group comparisons were evaluated by Student’s *t* test for parametric data and the Mann‒Whitney U test for nonparametric data. The Kruskal–Wallis test was used for nonparametric continuous parameters, and the chi-square test was used for categorical parameters to compare the differences between two or more groups. Post hoc tests (Bonferroni, Dunn) were used to determine which paired group was statistically significant. Correlations between continuous data were examined with the Spearman correlation test. A *p* value of <0.05 was considered to indicate statistical significance.

## 3. Results

### 3.1. Sociodemographic Characteristics

The study included 42 parents of children with SMA and 42 parents of children without chronic diseases. The median age and sex ratios were comparable between the patient and control groups. The median number of siblings was higher in the SMA group [1 (0–2)] compared to the control group [0 (0–1)] (*p* = 0.037). The parents of children with SMA were more likely to be employed as workers (40.5%) and less likely to be unemployed (21.4%) (*p* = 0.002). The proportion of low-income families was 26.2% higher in the SMA group, while the proportion of medium- to high-income families was 11.9% lower compared to the control group (*p* < 0.001). Additionally, the rate of primary education was 35.7% higher, and university education was 26.2% lower among parents of children with SMA compared to the control group (*p* < 0.001) (Table 1).

### 3.2. Comparison of Scale Scores Between the Case and the Control Group

The median WHOQOL-BREF physical health score was 53.5 (IQR: 42.8–60.7) in parents of children with SMA and 58.9 (IQR: 50.0–65.1) in the control group (*p* = 0.004). Significant differences were also observed in the psychological health (63.1 ± 17.2 vs. 71.9 ± 12.1; *p* = 0.009), social relationships (59.1 ± 22.0 vs. 71.2 ± 17.4; *p* = 0.007), and environmental (54.6 [IQR: 39.8–75.0] vs. 71.8 [IQR: 60.9–84.3]; *p* < 0.001) domains of the WHOQOL-BREF. Likewise, the median HADS depression score was significantly higher in the SMA group (9.0 [IQR: 4.0–12.0]) than in the control group (5.5 [IQR: 3.7–8.0]; *p* = 0.005). All of these differences were statistically significant. In contrast, no significant differences were found between the groups in the ZCBS, SWLS, or Flourishing Scale scores (*p* > 0.005) (Table 2).

### 3.3. Comparison of Scale Scores Between SMA Types

The median ZCBS score was significantly higher for SMA type 1 patients [35 (29–41.5)] compared to SMA type 3 patients [15 (11.5–30)] (*p* = 0.010). Similarly, the median HADS anxiety score was higher for SMA type 1 patients [10 (6.5–13)] than for SMA type 3 patients [4 (2.5–10)] (*p* = 0.037). The WHOQOL-BREF subscale values, SWLS scores, and the median Flourishing Scale scores showed no significant differences across SMA types (*p* > 0.005) (Table 3).

### 3.4. The Correlational Analysis Between the Scale Scores in the Case Group

Table 4 presents the correlations between the scales in the case group. A weak negative correlation was found between the environmental score and the ZCBS score (*p* = 0.003), whereas other WHOQOL-BREF subscales showed no significant association with the ZCBS (*p* > 0.005). The HADS anxiety and HADS depression subscales showed weak negative correlations with the WHOQOL-BREF subscales (*p* < 0.005). Moderate positive correlations were observed between the HADS anxiety subscale and the ZCBS scores (*p* < 0.001) and between the HADS anxiety and HADS depression subscales (*p* < 0.001). The HADS depression subscale and ZCBS scores demonstrated a weak positive correlation (*p* < 0.001). The SWLS scores correlated weakly with the physical, social, and environmental subscales and moderately with the psychological subscale (*p* < 0.005). Finally, weak negative correlations were found between the anxiety and depression subscales and SWLS scores (*p* < 0.005) and between the Flourishing Scale and the SWLS (*p* = 0.021).

## 4. Discussion

This study is among the first to compare the health-related quality of life (HRQOL), caregiver burden, symptoms of depression and anxiety, life satisfaction, and well-being in parents of children with SMA to those of parents of healthy controls. All HRQOL subdomains were found to be lower, and symptoms of depression were higher in the case group. However, no significant differences were observed between the groups in terms of caregiver burden, symptoms of anxiety, life satisfaction, or mental well-being. Additionally, caregiver burden and symptoms of anxiety were higher in parents of children with SMA type 1 compared to those with SMA types 2 and 3. HRQOL in parents was negatively correlated with symptoms of depression and anxiety, but no significant association was found between caregiver burden and most HRQOL subdomains. Consequently, our hypotheses were partially confirmed.

### 4.1. Comparison of Case and Controls Groups

In our results, the parents of children diagnosed with SMA exhibited greater impairments in physical health, psychological health, social relationships, and environmental quality of life domains compared to the parents of healthy controls, which is consistent with previous findings. Research involving caregivers of individuals with muscular dystrophies has similarly shown that their HRQOL is lower than national population averages [31]. In line with our findings, several studies investigating HRQOL in caregivers of individuals with SMA have reported significant impairments in caregivers’ quality of life [21,32,33]. However, one study from Germany found that caregivers’ HRQOL was comparable to that of the general population [21]. Taken together, these findings suggest that parents of children with SMA may require targeted support to improve outcomes across all HRQOL subdomains.

The ZCBS has been widely used in the assessment of caregiver burden among parents of children with SMA [32,33,34,35,36]. Similar to the previous studies, in our sample the parents of children with SMA were found to have mild to moderate levels of caregiver burden. Nevertheless, contrary to our initial hypothesis, caregiver burden was not significantly higher than the control group. This finding suggests that caregiving for a healthy child may also involve a degree of burden, and that having a child diagnosed with SMA—regardless of subtype—may not necessarily increase caregiver burden.

Symptoms of depression and anxiety were notably elevated in our case group. However, while anxiety symptoms did not differ significantly between groups, depression symptoms were meaningfully higher in the case group. Furthermore, the mean depression scores exceeded established cutoff values, indicating clinically significant levels of depressive symptoms among parents of children with SMA. These elevated levels suggest potential functional impairments, emphasizing the need for clinical assessment and possible intervention [19]. Consistently, our findings on health-related quality of life (HRQOL) demonstrated substantial impairment across all domains compared to typical populations. Previous studies have similarly reported clinically elevated levels of depression and anxiety in parents of children with SMA [37,38]. On the other hand, one study found that depression and anxiety symptoms in this population remained below clinical thresholds [21]. Taken together, these findings suggest that while a diagnosis of SMA in children may contribute to increased depressive symptoms in parents, various factors may influence anxiety levels. One such factor, the type of SMA—which provides insights into the severity and progression of the disease—has been investigated and will be discussed in this study. Additionally, it is important to note that all children in the case group were undergoing nusinersen treatment, and no conclusions can be drawn regarding newly diagnosed children or those not receiving treatment.

In our sample, the parents of children with SMA reported life satisfaction levels below a moderate level, with no significant difference in life satisfaction between the groups. Cremers et al. reported that mothers of individuals with SMA were “satisfied” or “very satisfied” with their activities [10]. Similar findings have been reported in the literature for other pediatric-onset chronic illnesses, such as multiple sclerosis and cancer [37,38]. Growing evidence suggests that caregiving can be experienced positively, even under conditions of high burden [39,40,41]. In our sample, this slight dissatisfaction appears to be consistent with the findings of other studies conducted in our country on caregivers of patients requiring chronic care [42,43]. Additionally, factors such as whether the illness is progressive or stable, or the presence of hope for treatment, may shape parents’ perceptions of their caregiving experience [44]. In this context, it can be suggested that, despite its challenging aspects, the process of supporting their children may be partially fulfilling for parents of children with SMA, similar to the experience reported by parents of healthy children.

### 4.2. Comparison of SMA Caregivers by Disease Type

According to our results, the caregiver burden scores and HADS anxiety scores were significantly higher in the parents of children with SMA type 1 compared to those of children with SMA type 3. This finding aligns with previous research reporting higher stress levels among parents of individuals with SMA types 1 and 2 [45]. Similarly, our results are consistent with the study by Chambers [46], which found reduced HRQOL among caregivers of children with SMA, although no differences were observed between SMA subtypes. On the other hand, a recent study conducted in our country reported no significant differences in the anxiety scores across SMA subtypes [36]. However, it was stated that the number of children with SMA type 3 included in the study was limited. Based on these findings, we can suggest that SMA type is a significant factor for caregiver burden and symptoms of parental anxiety during nusinersen treatment. In addition, parents of children with SMA type 1, which is associated with a more severe clinical profile and poorer prognosis, may experience greater levels of psychological burden and anxiety.

### 4.3. Relationships Between the HRQOL, Caregiver Burden, and Symptoms of Depression and Anxiety in the Case Group

In SMA, increased levels of dependency and the need for long-term care can lead to significant stress and burden on caregivers [8]. Although low quality of life has been considered one of the indicators of caregiver burden in some studies [11], our findings revealed a correlation only between the environmental domain of HRQOL and caregiver burden. As SMA is a disease characterized by physical disability, aspects of the environmental domain, such as freedom, home environment, and transportation, may be more significantly affected. Various factors, including personality, cognitive functioning, and cultural influences, may also impact health-related quality of life (HRQOL) in muscular dystrophies [47]. However, not all of these factors were examined in this study, and therefore, no conclusions can be drawn regarding their contribution to HRQOL. Nevertheless, we suggest that future research should explore the role of psychosocial dynamics and contextual factors in the impairment of HRQOL among parents of children with spinal muscular atrophy (SMA).

Our findings also show that high levels of anxiety and depressive symptoms in caregivers are associated with lower quality of life and life satisfaction. Although we did not identify any studies investigating this relationship in parents of children with SMA, a study on parents of children with epilepsy has shown an inverse relationship between quality of life and internalization, which aligns with our findings [48]. This finding highlights the importance of assessing caregivers for mental health symptoms. Finally, our correlation analysis revealed a moderate positive correlation between the HADS anxiety subscale score and ZCBS score. Similarly, Wohnrade and colleague found that increased caregiver burden was associated with higher levels of anxiety and dissatisfaction. Additionally, it has been demonstrated that the HADS anxiety subscale is a predictor of caregiver burden [21]. Similarly, studies on caregivers have shown a strong relationship between caregiver burden and anxiety symptoms [49]. Therefore, in line with the literature, our findings suggest that among the factors examined in this study, symptoms of anxiety emerge as a prominent factor associated with the increased caregiver burden. Consequently, we recommend addressing the symptoms of depression and anxiety, along with caregiver burden, in the management of low health-related quality of life (HRQOL) among parents of children with SMA. Psychoeducational interventions and psychiatric consultation for parents can be recommended, considering that a significant proportion may exhibit symptoms of depression and anxiety that require treatment. Additionally, the impairment observed across all domains of HRQOL highlights the need for the integration of social services into the management team.

### 4.4. Strengths and Limitations

This study is the first to compare parents of children with SMA and parents of healthy controls in terms of parental quality of life. Although SMA is a rare disease, we included a similar number of children diagnosed with SMA types 1, 2, and 3, allowing comparisons across subtypes. In addition, the sample featured a balanced representation of mothers and fathers among the participants. However, this study has several limitations. First, it was a single-center study conducted with a relatively small sample size. Multi-center studies with larger samples could provide a more comprehensive understanding of the challenges faced by parents of children with SMA. Second, the lower socioeconomic status observed in the case group may have influenced the findings. Finally, the fact that all children with SMA in this study were receiving nusinersen treatment may have contributed to more positive parental perceptions, potentially influencing treatment expectations and the study outcomes. Therefore, future research involving newly diagnosed children or those who have not yet initiated treatment may offer valuable insights into the impact on parents. Furthermore, longitudinal studies are needed to better elucidate the impact of the disease at various stages, providing a more comprehensive understanding over time. Finally, it is recommended that future studies conduct an in-depth examination of factors potentially associated with HRQOL, such as personality traits, coping strategies, stigmatization, and cultural determinants.

## 5. Conclusions

This study underscores the deterioration in all aspects of quality of life among parents of children with SMA, aligning with findings in the existing literature. Psychiatric evaluation may be recommended for these parents, as having a child with SMA was found to be associated with increased symptoms of depression in this study. Furthermore, the results indicate that parents of children with SMA type 1 may require additional support to address elevated levels of caregiver burden and anxiety.

## Figures and Tables

**Table 1:** Comparison of children and caregiver characteristics with those of the control.

Characteristics *n* (%)	SMA Group (*n* = 42)	Control Group (*n* = 42)	*p* Value
Age ^1^	7 (5–12)	7 (5–12)	0.999 ^a^
Gender ^1^			0.383 ^b^
Female	23 (54.8)	19 (45.2)	
Male	19 (45.2)	23 (54.8)	
Number of siblings ^1^	1 (0–2)	0 (0–1)	**0.037 ^a^**
Lives in Istanbul ^1^			0.463 ^b^
No	13 (31)	10 (23.8)	
Yes	29 (69)	32 (76.2)	
Going to school ^1^			**<0.001 ^b^**
No	28 (66.7)	9 (21.4)	
Yes	14 (33.3)	33 (78.6)	
Caregiver			0.999 ^b^
Mother	22 (52.4)	22 (52.4)	
Father	20 (47.6)	20 (47.6)	
Caregiver			**0.002 ^b^**
Unemployed	16 (38.1)	9 (21.5)	
Worker	17 (40.5)	8 (19)	
Officer	9 (21.4)	25 (59.5)	
Known medical diagnosis ^1^			0.786 ^b^
No	33 (78.6)	34 (81)	
Yes	9 (21.4)	8 (19)	
Psychiatric diagnosis ^1^			0.999 ^b^
No	36 (85.7)	36 (85.7)	
Yes	6 (14.3)	6 (14.3)	
Regular medication ^1^ use			0.192 ^b^
No	35 (83.3)	30 (71.4)	
Yes	7 (16.7)	12 (28.6)	
Income Status ^2^			**<0.001 ^b^**
Very low	11 (26.2)	1 (2.4)	
Low	26 (61.9)	22 (52.4)	
Moderate/high	5 (11.9)	19 (45.2)	
Level of education ^2^			**<0.001 ^b^**
Primary education	15 (35.7)	4 (9.5)	
High School	16 (38.1)	8 (19)	
University	11 (26.2)	30 (71.5)	
Marital Status ^2^			0.332 ^b^
Single	7 (16.7)	4 (9.5)	
Married	35 (83.3)	38 (90.5)	

^1^: Children with SMA, ^2^: Caregiver, ^a^ Mann–Whitney U Test, ^b^ Chi-square test.

**Table 2:** Comparison of scale scores between children with SMA and the control group.

Parameters	SMA Group (*n* = 42)	Control Group (*n* = 42)	*p* Value
Physical health QoL	53.5 (42.8–60.7)	58.9 (50–65.1)	**0.004 ^a^**
Psychological health QoL	63.1 ± 17.2	71.9 ± 12.1	**0.009 ^b^**
Social relationships QoL	59.1 ± 22	71.2 ± 17.4	**0.007 ^b^**
Environmental QoL	54.6 (39.8–75)	71.8 (60.9–84.3)	**<0.001 ^a^**
ZCBS	31.5 (15.7–40)	26.5 (18–37.2)	0.623 ^a^
HADS Anxiety Subscale	8.5 (4.7–11)	8 (5–11)	0.802 ^a^
HADS Depression Subscale	9 (4–12)	5.5 (3.7–8)	**0.005 ^a^**
SWLS	15 (9–19)	16 (11–19.2)	0.251 ^a^
The Flourishing Scale	40.5 (31.7–49)	43.5 (29.7–48)	0.929 ^a^

ZCBS: The Zarit Caregiver Burden Scale, HADS: The Hospital Anxiety and Depression Scale, SWLS: The Satisfaction with Life Scale, ^a^ Mann–Whitney U Test, ^b^ Student *t* Test.

**Table 3:** Comparison of scale scores between SMA types.

Parameters	Type I (*n* = 16)	Type II (*n* = 13)	Type III (*n* = 13)	*p* Value
Physical health QoL	50 (42.8–57.1)	57.1 (44.6–64.2)	50 (32.1–66)	0.391
Psychological health QoL	54.1 (50–61.4)	70.8 (62.5–79.1)	62.5 (45.8–85.4)	0.061
Social relationships QoL	54.1 (37–72.9)	58.3 (45.8–83.3)	58.3 (45.8–70.8)	0.658
Environmental QoL	53.1 (37–60.9)	50 (37.5–73.4)	62.5 (46.8–81.2)	0.317
ZCBS	35 (29–41.5)	31 (18–46)	15 (11.5–30)	**0.010**
HADS Anxiety Subscale	10 (6.5–13)	8 (5–9)	4 (2.5–10)	**0.037**
HADS Depression Subscale	9.5 (5.2–13.7)	8 (5–11.5)	7 (3–10)	0.367
SWLS	15.5 (10–17)	15 (7.5–19.5)	14 (9–20)	0.996
The Flourishing Scale	37 (31.2–44.2)	40 (31–50.5)	44 (32–50.5)	0.510

ZCBS: The Zarit Caregiver Burden Scale, HADS: The Hospital Anxiety and Depression Scale, SWLS: The Satisfaction with Life Scale, *p* value: Kruskal‒Wallis Test.

**Table 4:** The correlation between scale scores in children with SMA.

Parameters		Psychological Health QoL	Social Relationships QoL	Environmental QoL	ZCBS	HADS Anxiety Subscale	HADS Depression Subscale	SWLS	The Flourishing Scale
Physical health QoL	r	0.681	0.589	0.528	−0.292	−0.345	−0.430	0.414	−0.056
	*p*	**<0.001**	**<0.001**	**<0.001**	0.061	**0.025**	**0.004**	**0.006**	0.725
Psychological health QoL	r	1.000	0.553	0.593	−0.285	−0.410	−0.581	0.597	−0.191
	*p*		**<0.001**	**<0.001**	0.067	**0.007**	**<0.001**	**<0.001**	0.225
Social relationships QoL	r		1.000	0.676	−0.297	−0.442	−0.560	0.430	0.012
	*p*			**<0.001**	0.056	**0.003**	**<0.001**	**0.005**	0.939
Environmental QoL	r			1.000	−0.451	−0.431	−0.556	0.419	−0.143
	*p*				**0.003**	**0.004**	**<0.001**	**0.006**	0.367
ZCBS	r				1.000	0.632	0.400	−0.219	0.027
	*p*					**<0.001**	**0.009**	0.163	0.865
HADS Anxiety Subscale	r					1.000	0.635	−0.325	0.032
	*p*						**<0.001**	**0.036**	0.838
HADS Depression Subscale	r						1.000	−0.476	0.296
	*p*							**0.001**	0.057
SWLS	r							1.000	−0.354
	*p*								**0.021**

Spearman Correlation (<0.25 very weak; 0.26–0.49 weak; 0.50–0.69 moderate; 0.70–0.89 high; 0.90–1.0 very high). ZCBS: The Zarit Caregiver Burden Scale, HADS: The Hospital Anxiety and Depression Scale, SWLS: The Satisfaction with Life Scale.

## Data Availability

The original contributions presented in this study are included in the article. Further inquiries can be directed to the corresponding author.

## References

[1] The World Health Organization WHOQOL User Manual. https://www.who.int/publications/i/item/WHO-HIS-HSI-Rev.2012.03.

[2] Macedo E.C., Da Silva L.R., Paiva M.S., Ramos M.N.P. (2015). Burden and Quality of Life of Mothers of Children and Adolescents with chronic Illnesses: An Integrative Review. Rev. Lat. Am. Enferm..

[3] Ransmayr G. (2021). Challenges of Caregiving to Neurological Patients. Wien. Med. Wochenschr..

[4] Nishio H., Niba E.T.E., Saito T., Okamoto K., Takeshima Y., Awano H. (2023). Spinal Muscular Atrophy: The Past, Present, and Future of Diagnosis and Treatment. Int. J. Mol. Sci..

[5] Younger D.S., Mendell J.R. (2023). Childhood Spinal Muscular Atrophy. Handbook of Clinical Neurology.

[6] Mercuri E., Sumner C.J., Muntoni F., Darras B.T., Finkel R.S. (2022). Spinal Muscular Atrophy. Nat. Rev. Dis. Primers.

[7] Cooper K., Nalbant G., Sutton A., Harnan S., Thokala P., Chilcott J., McNeill A., Bessey A. (2024). Systematic Review of Newborn Screening Programmes for Spinal Muscular Atrophy. Int. J. Neonatal Screen..

[8] Brandt M., Johannsen L., Inhestern L., Bergelt C. (2022). Parents as Informal Caregivers of Children and Adolescents with Spinal Muscular Atrophy: A Systematic Review of Quantitative and Qualitative Data on the Psychosocial Situation, Caregiver Burden, and Family Needs. Orphanet J. Rare Dis..

[9] Lee Y.J., Kim A.R., Lee J.M., Shim Y.K., Cho J.S., Ryu H.W., Kwon S., Chae J.H. (2023). Impact of Nusinersen on the Health-Related Quality of Life and Caregiver Burden of Patients with Spinal Muscular Atrophy with Symptom Onset after Age 6 Months. Muscle Nerve.

[10] Cremers C.H., Fischer M.J., Kruitwagen-van Reenen E.T., Wadman R.I., Vervoordeldonk J.J., Verhoef M., Visser-Meily J.M., van der Pol W.L., Schröder C.D. (2019). Participation and Mental Well-Being of Mothers of Home-Living Patients with Spinal Muscular Atrophy. Neuromuscul. Disord..

[11] Landfeldt E., Abner S., Pechmann A., Sejersen T., McMillan H.J., Lochmüller H., Kirschner J. (2023). Caregiver Burden of Spinal Muscular Atrophy: A Systematic Review. Pharmacoeconomics.

[12] Munsat T.L., Davies K.E. (1992). International SMA Consortium Meeting. (26–28 June 1992, Bonn, Germany). Neuromuscul. Disord..

[13] Harper A., Power M., Orley J., Herrman H., Schofield H., Murphy B., Metelko Z., Szabo S., Pibernik-Okanovic M., Quemada N. (1998). Development of the World Health Organization WHOQOL-BREF Quality of Life Assessment. The WHOQOL Group. Psychol. Med..

[14] Eser E., Fidaner H., Fidaner C., Eser S.Y., Elbi H., Göker E. (1999). WHOQOL-100 ve WHOQOL-BREF’in psikometrik özellikleri. Psikiyatr. Psikol. Psikofarmakol. (3p) Derg..

[15] Błauciak M., Ubysz J., Pokryszko-Dragan A., Koszewicz M. (2024). The Impact of Comorbidities and Motor Impairment on the Quality of Life of Patients with Spinal Muscular Atrophy: A Case-Control Study. J. Clin. Med..

[16] Pugh J.D., McCoy K., Williams A.M., Pienaar C.A., Bentley B., Monterosso L. (2022). Neurological Patient and Informal Caregiver Quality of Life, and Caregiver Burden: A Cross-Sectional Study of Postdischarge Community Neurological Nursing Recipients. Contemp. Nurse.

[17] Zarit S.H., Reever K.E., Bach-Peterson J. (1980). Relatives of the Impaired Elderly: Correlates of Feelings of Burden. Gerontologist.

[18] Özlü A., Yıldız M., Aker A.T. (2009). A Reliability and Validity Study on the Zarit Caregiver Burden Scale. Arch. Neuropsychiatry.

[19] Aydemir O., Güvenir T., Küey L., Kültür S. (1997). Reliability and Validity of the Turkish Version of Hospital Anxiety and Depression Scale. Turk. J. Psychiatry.

[20] Zigmond A.S., Snaith R.P. (1983). The Hospital Anxiety and Depression Scale. Acta Psychiatr. Scand..

[21] Wohnrade C., Velling A.K., Mix L., Wurster C.D., Cordts I., Stolte B., Zeller D., Uzelac Z., Platen S., Hagenacker T. (2023). Health-Related Quality of Life in Spinal Muscular Atrophy Patients and Their Caregivers—A Prospective, Cross-Sectional, Multi-Center Analysis. Brain Sci..

[22] Burke T., Hardiman O., Pinto-Grau M., Lonergan K., Heverin M., Tobin K., Staines A., Galvin M., Pender N. (2018). Longitudinal Predictors of Caregiver Burden in Amyotrophic Lateral Sclerosis: A Population-Based Cohort of Patient–Caregiver Dyads. J. Neurol..

[23] Diener E., Emmons R.A., Larsem R.J., Griffin S. (1985). The Satisfaction with Life Scale. J. Pers. Assess..

[24] Chu S.Y., Tan C.L. (2019). Perception on the Quality of Life, Communication and Life Satisfaction among Individuals with Parkison’s and Their Caregivers. Ethiop. J. Health Sci..

[25] Leibach G.G., Stern M., Arelis A.A., Islas M.A.M., Barajas B.V.R. (2016). Mental Health and Health-Related Quality of Life in Multiple Sclerosis Caregivers in Mexico. Int. J. MS Care.

[26] Dağlı A., Baysal N. (2016). Adaptation of the Satisfaction with Life Scale into Turkish: The Study of Validity and Reliability. Electron. J. Soc. Sci..

[27] Arrindell W.A., Meeuwesen L., Huyse F.J. (1991). The Satisfaction with Life Scale (SWLS): Psychometric Properties in a Non-Psychiatric Medical Outpatients Sample. Pers. Individ. Differ..

[28] Kusier A.O., Folker A.P. (2021). The Satisfaction with Life Scale: Philosophical Foundation and Practical Limitations. Health Care Anal..

[29] Telef B.B. (2013). The Adaptation of Psychological Well-Being into Turkish: A Validity and Reliability Study. Hacet. Üniv.-J. Educ..

[30] Diener E., Napa Scollon C., Lucas R.E. (2009). The Evolving Concept of Subjective Well-Being: The Multifaceted Nature of Happiness. Assessing Well-Being.

[31] Domaradzki J., Walkowiak D. (2024). Quality of Life and Caregiving Burden Associated with Parenting a Person with Duchenne/Becker Muscular Dystrophy in Poland. Orphanet J. Rare Dis..

[32] Peña-Longobardo L.M., Aranda-Reneo I., Oliva-Moreno J., Litzkendorf S., Durand-Zaleski I., Tizzano E., López-Bastida J. (2020). The Economic Impact and Health-Related Quality of Life of Spinal Muscular Atrophy. An Analysis across Europe. Int. J. Environ. Res. Public Health.

[33] López-Bastida J., Peña-Longobardo L.M., Aranda-Reneo I., Tizzano E., Sefton M., Oliva-Moreno J. (2017). Social/Economic Costs and Health-Related Quality of Life in Patients with Spinal Muscular Atrophy (SMA) in Spain. Orphanet J. Rare Dis..

[34] Acar A.E., Saygı E.K., İmamoğlu S., Öztürk G., Ünver O., Ergenekon P., Gökdemir Y., Özel G., Türkdoğan D. (2021). The Burden of Primary Caregivers of Spinal Muscular Atrophy Patients and Their Needs. Turk. Arch. Pediatr..

[35] Du L., Dong H., Miao C., Jia F., Shan L. (2022). Analysis of Scores of Symptom Checklist 90 (SCL-90) Questionnaire of 182 Parents of Children with Spinal Muscular Atrophy: A Cross-Sectional Study. Transl. Pediatr..

[36] Ergenekon A.P., Gümüş Z., Yegit C.Y., Cenk M., Gulieva A., Kalyoncu M., Selcuk M., Karabulut S., Ozturk G., Eralp E.E. (2023). Depression, Anxiety, and Sleep Quality of Caregivers of Children with Spinal Muscular Atrophy. Pediatr. Pulmonol..

[37] Hamama L., Hamama-Raz Y., Lebowitz-Sokolover K., Ganelin-Cohen E. (2024). Well-Being among Parents of Youth with Multiple Sclerosis: A Preliminary Longitudinal Study. Front. Psychol..

[38] Pozo Muñoz C., Bretones Nieto B., Vázquez López M.Á. (2021). When Your Child Has Cancer: A Path-Analysis Model to Show the Relationships between Flourishing and Health in Parents of Children with Cancer. Int. J. Environ. Res. Public Health.

[39] Anderson E.W., White K.M. (2018). “It Has Changed My Life”: An Exploration of Caregiver Experiences in Serious Illness. Am. J. Hosp. Palliat. Care.

[40] Manalel J.A., Sumrall S., Davidson H., Grewal M., Granovetter M.A., Koehly L.M. (2024). Stress, Coping, and Positive Aspects of Caregiving among Caregivers of Children with Rare Disease. Psychol. Health.

[41] Pristavec T., Pruchno R. (2018). The Burden and Benefits of Caregiving: A Latent Class Analysis. Gerontologist.

[42] Sezek I., Cubukcu M., Muderrısoglu S. (2023). Care Burden and Life Satisfaction of Caregivers Who Are Providing Home Health Care to Patients. Risk Manag. Healthc Policy.

[43] Özcan F., Gürçay E., Kalem Özgen A.N., Demir Y. (2024). Outcomes and Predictors of Stress among Turkish Family Caregivers of Patients with Acquired Brain Injury. Appl. Neuropsychol. Adult..

[44] Ho M., Liang R., Ip Y., Zhi H., Wong W., Chan H. (2021). The Impact of Paediatric Neuromuscular Disorders on Parents’ Health-Related Quality of Life and Family Functioning. HK J. Paediatr. (New Ser.).

[45] Von Gontard A., Rudnik-Schöneborn S., Zerres K. (2012). Stress and Coping in Parents of Children and Adolescents with Spinal Muscular Atrophy. Klin. Padiatr..

[46] Chambers G.M., Settumba S.N., Carey K.A., Cairns A., Menezes M.P., Ryan M., Farrar M.A. (2020). Prenusinersen Economic and Health-Related Quality of Life Burden of Spinal Muscular Atrophy. Neurology.

[47] Bann C.M., Abresch R.T., Biesecker B., Conway K.C., Heatwole C., Peay H., Scal P., Strober J., Uzark K., Wolff J. (2015). Measuring Quality of Life in Muscular Dystrophy. Neurology.

[48] Puka K., Tavares T.P., Anderson K.K., Ferro M.A., Speechley K.N. (2018). A Systematic Review of Quality of Life in Parents of Children with Epilepsy. Epilepsy Behav..

[49] del-Pino-Casado R., Priego-Cubero E., López-Martínez C., Orgeta V. (2021). Subjective Caregiver Burden and Anxiety in Informal Caregivers: A Systematic Review and Meta-Analysis. PLoS ONE.

